# Serum Concentrations of TNF *α* and Its Soluble Receptors in Patients with Adrenal Tumors Treated by Surgery

**DOI:** 10.3390/ijms11062281

**Published:** 2010-05-26

**Authors:** Jan Komorowski, Jolanta Jurczynska, Tomasz Stepien, Krzysztof Kolomecki, Krzysztof Kuzdak, Henryk Stepien

**Affiliations:** 1 Department of Clinical Endocrinology, First Chair of Endocrinology, Medical University of Lodz, Sterlinga1/3, 91-425 Lodz, Poland; 2 Department of Endocrine and General Surgery, First Chair of Endocrinology, Pabianicka 62, Medical University of Lodz, 93-513 Lodz, Poland; 3 Department of Immunoendocrinology, First Chair of Endocrinology, Medical University of Lodz, Sterlinga1/3, 91-425 Lodz, Poland

**Keywords:** adrenal tumors, TNF α, TNF α R1, TNF α R2, adrenalectomy

## Abstract

The peripheral blood levels of TNF *α* and its soluble receptors were studied in 39 patients with malignant and benign adrenal tumors treated by adrenalectomy. The concentrations of TNF *α* were significantly elevated in patients with malignant tumors of the adrenal cortex and in patients with Conn’s syndrome compared to control. In patients with non-functioning adenomas and pheochromocytomas, TNF *α* levels were similar to those detected in the control. In subjects with myelolipomas, the serum concentration of TNF *α* was lower compared to the control. After adrenalectomy, the levels of TNF *α* were decreased in patients with malignant tumors and in patients with Conn’s syndrome, nonfunctioniong adenomas and pheochromocytomas compared to the concentration before surgery. The serum concentrations of soluble receptors of TNF *α* did not differ among different patient groups and compared to the control. After adrenalectomy, the blood concentrations of TNF *α* R1 and TNF *α* R2 were decreased in patients with Conn’s syndrome. However, to confirm practicality of the evaluation of TNF *α* and its soluble receptors in differential diagnosis in patients with adrenal tumors, a larger study group is needed.

## 1. Introduction

Modern imaging modalities lead to frequent detection of adrenal masses. Most of them are incidental findings. Although the majority of adrenocortical and adrenomedullary tumors are benign, there are no clinical and laboratory markers to distinguish most of them from malignant tumors [1,2]. The molecular mechanisms underlying the pathogenesis of these tumors have been recently unraveled. Because prognoses and surgical methods for adrenocortical adenomas and adrenal carcinomas are vastly different it is important to accurately differentiate the two tumor types. However, the study on the serum concentration of tumor necrosis factor *α* (TNF *α*) and its soluble receptors (TNF *α* R1 and TNF *α* R2) have not been performed in patients with adrenal tumors so far.

TNF *α* is a pleiotropic cytokine that plays a central role in inflammation and apoptosis. It modifies the inflammatory reactions and immune reactions in response to injury and infection [3]. TNF *α* plays also a necessary and beneficial role as mediator of host resistance to infection and tumor formation. Moreover, overproduction of TNF *α* or its overexpression can lead to a variety of pathologic conditions including wasting, systemic toxicity and septic shock. TNF *α* acts via two types of receptors (TNF *α* R1, TNF *α* R2), located on the cell surface. Type one of the TNF *α* receptor is involved in apoptosis. The physiological role of the type two TNF *α* receptor is unknown, but this type of receptor is also probably involved in apoptosis [4,5]. The soluble forms of these receptors arise as a result of shedding of the extracellular domain of the receptors and are present in low concentrations in serum and urine of healthy subjects [6,7].

The aim of the study was to evaluate the serum concentrations of TNF *α* and its soluble receptors in patients with malignant and benign adrenal tumors treated by adrenalectomy. We evaluated usefulness of these peptides as the laboratory markers in differential diagnosis.

## 2. Materials and Methods

The study group was composed of 39 subjects [28 females aged 25–82 (53.68 ± 16.60; *x* ± SD) and 11 males aged 46–74 (56.45 ± 7.55)]. Seven patients with malignant cortical tumors, seven patients with Conn’s syndrome, 10 patients with non-functioning adrenal adenomas, 12 patients with pheochromocytomas and three patients with myelolipomas (according to pathology data) were studied. The clinical diagnosis of the malignant tumors was suspected in patients with weight loss, high serum concentration of dehydroepiandrosterone sulfate and irregular, large (usually with a diameter greater than 6 cm) mass found by computer tomography imaging. The diagnosis of the Conn’s syndrome was based on elevated level of plasma aldosterone, low level of plasma renin and an aldosterone/renin ratio of greater than 5.4. The diagnosis of pheochromocytoma was suspected in patients with poorly controlled hypertension associated with bouts of sweating, headache, palpitations, a high serum concentration of chromogranin A and a high level of metanephrins in urine. In patients with nonfunctioning adenomas and myelolipomas, the tumors were incidentally found during computer tomography imaging.

The adrenal tumors were surgically removed. All the investigations were performed one day prior to surgical treatment and one month after adrenalectomy. The clinical diagnosis was confirmed by histological investigation.

The control group was composed of 10 healthy subjects (six females and four males) aged 36–60 (49.5 ± 8.38). The presence of adrenal tumor was excluded on the basis of physical examination, laboratory tests and ultrasound examination of the abdomen. All patients and control group were included in the study only after giving informed consent. The study was approved by the Local Ethical Committee.

The concentration of TNF *α* and its soluble receptors were measured by enzyme immunoassay (ELISA) (R&D Systems, USA). The intra-assay precision for TNF *α* was 4.67%, for TNF *α* R1 – 4.43%, and for TNF *α* R2 was 3.53%. The inter - assay precision for TNF *α* was 5.8%, for TNF *α* R1 – 6.1%, and for TNF *α* R2 was 4.07%. The statistical analysis was performed using the non - parametric Kruskal-Wallis test and Wilxocon test. A *p*-value of less than 0.05 was considered statistically significant.

## 3. Results

**Figure 1.**
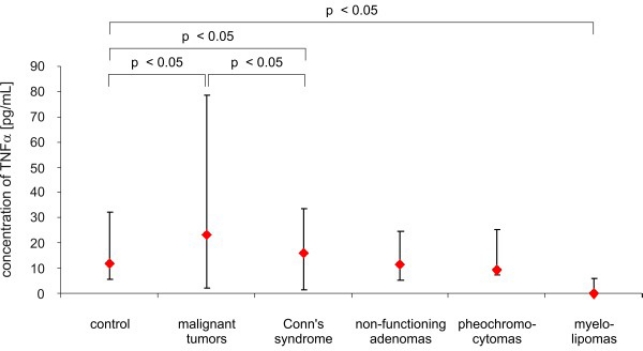
Concentrations of TNF *α* (pg/mL) in peripheral blood of patients and control (median and range).

**Figure 2.**
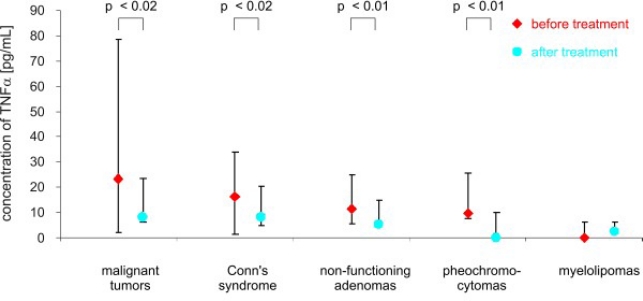
Concentrations of TNF *α* (pg/mL) in peripheral blood studied groups of patients before and after surgery (median and range).

**Figure 3.**
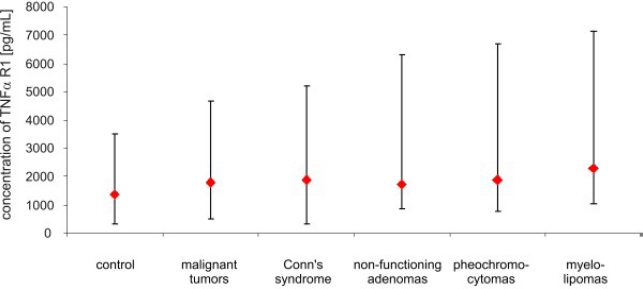
Concentrations of TNF R1 *α* (pg/mL) in peripheral blood of studied groups of patients and control (median and range).

**Figure 4.**
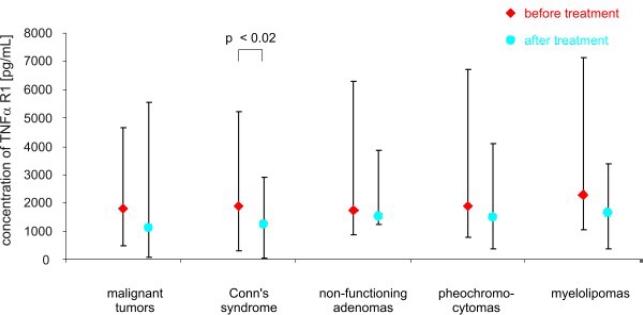
Concentrations of TNF R1 *α* (pg/mL) in peripheral blood of studied groups of patients before and after surgery (median and range).

**Figure 5.**
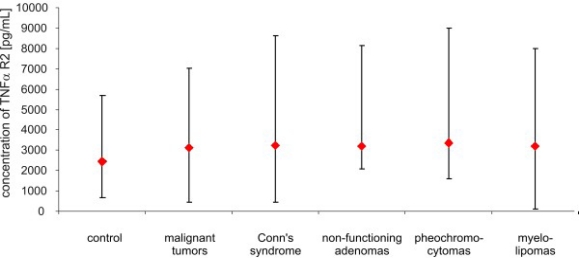
Concentrations of TNF R2 *α* (pg/mL) in peripheral blood of studied groups of patients and control (median and range).

**Figure 6.**
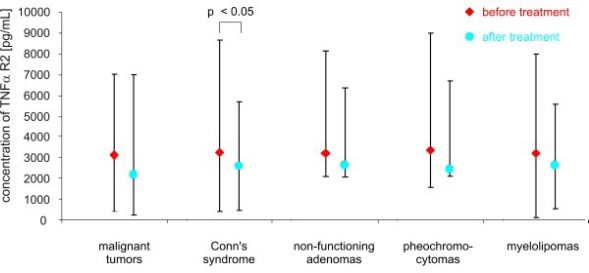
Concentrations of TNF R2 *α* (pg/mL) in peripheral blood of studied groups before and after surgery (median and range).

The concentrations of TNF *α*, TNF *α* R1 and TNF *α* R2 before surgery and after surgery are presented in Figures 1–6. The mean (*x* ± SD) concentration of TNF *α* in the control group was 12.09 ± 4.31 pg/mL, the mean concentration of TNF *α* R1 was 1,479.2 ± 388.23 pg/mL, and the mean concentration of TNF *α* R2 was 2,456.7 ± 521.12 pg/mL. The concentration of TNF *α* was significantly elevated in patients with malignant tumors of the adrenal cortex, compared to the control (30.04 ± 13.28 pg/mL *vs.* 12.09 ± 4.31 pg/mL; *p* < 0.05), and in patients with Conn’s syndrome, compared to the control (16.22 ± 1.11 pg/mL *vs.* 12.09 ± 4.31 pg/mL; *p* < 0.05). The highest levels of TNF *α* in patients with malignant tumors of the cortex were noted. In patients with non-functioning adenomas and pheochromocytomas, TNF *α* levels were not significantly different from those of the control, (*p* > 0.05) and were respectively 11.0 ± 2.03 pg/mL; 9.14 ± 4.58 pg/mL. In patients with myelolipomas, the serum concentration of TNF *α* was lower, compared to control (2.06 ± 3.58 pg/mL *vs.* 12.09 ± 4.31 pg/mL; *p* < 0.05) (Figure 1).

After adrenalectomy, the levels of TNF *α* were lower in patients with malignant tumors (8.60 ± 4.35 pg/mL *vs.* 30.4 ± 13.28 pg/mL; *p* < 0.02), and in patients with Conn’s syndrome (8.07 ± 3.03 pg/mL *vs.* 16.22 ± 1.11 pg/mL; *p* < 0.02), non-functioning adenomas (4.77 ± 3.91 pg/mL *vs.* 11.0 ± 2,03 pg/mL; *p* < 0.01) and pheochromocytomas (1.95 ± 3.23 pg/mL *vs.* 9.14 ± 4.58 pg/mL; *p* < 0.01) than before surgery (Figure 2). The serum concentration of soluble receptors of TNF *α* did not differ among different patient groups or the control (Figures 3 and 5). After adrenalectomy, the serum concentration of TNF *α* R1 and TNF *α* R2 decreased in patients with Conn’s syndrome (1365.86 ± 156.51 *vs.* 2309.43 ± 736.86 pg/mL; *p* < 0.02 and 2587.71 ± 406.81 pg/mL *vs.* 3652.14 ± 956.62 pg/mL; *p* < 0.05 respectively) (Figures 4 and 6).

No significant correlations between the size of the tumors and the serum level of TNF *α* and its receptors were found.

## 4. Discussion

TNF *α* is a transmembrane protein described originally as a protein which causes the haemorrhagic necrosis of experimental tumors [8]. Further studies revealed that TNF *α* possesses proinflammatory activity and plays a key role in apoptosis [9–12]. A low level of TNF *α* and its receptors may increase susceptibility to infections [13]. TNF *α* is also probably involved in development of lymphoid tissue [14].

The role of TNF *α* in pathogenesis of malignant tumors is not clearly understood. Mocellin and Nitti [15] suggest that TNF *α* may represent one of the molecular links between chronic inflammation and further development of malignant tumors. Leibovich *et al*. [16] revealed that TNF *α* may promote angiogenesis but others [17] did not confirm the proangiogenic activity of TNF *α*. Schweigerer *et al*. [17] assessed the influence of TNF *α* on the proliferation of endothelial cells derived from blood vessels of the brain and adrenal cortex. In both types of these cells, TNF *α* decreased the proliferative activity of endothelial cells.

TNF *α* acts also as a paracrinal and autocrinal regulator of the growth and development of the adrenocortical cells. Call *et al*. [18] revealed that bovine adrenal glands secrete TNF *α* and IL-6. They also proved, using immunoassay techniques, that the glomerular zone of the adrenal cortex is the site of the greatest synthesis of TNF *α*. The increased level of TNF *α* in our study in patients with Conn’s syndrome may confirm that the glomerular zone of the adrenal cortex mainly secretes TNF *α*. The secretion of TNF *α* by human adrenal glands confirmed the studies of González-Hernández *et al*. [19,20] and Päth *et al*. [21]. It is also known that TNF *α* has an influence on synthesis and secretion of steroid hormones in adrenal cortex [22]. Moreover, that elevated levels of TNF *α* occur in the blood of patients with malignant tumors. Cimino *et al*. [23] observed an increased level of TNF *α* (and also interleukin 2 and soluble forms of IL-2 receptors) in the blood of patients with acute leukemia. Elevated levels of TNF *α* were also confirmed in the peripheral blood of patients with melanoma [24], renal carcinoma [25] and pancreas carcinoma [26].

The results of our study showed the highest level of TNF *α* in patients with malignant tumors (primary adrenocortical carcinoma and metastatic tumors). It may be due to activation of the immunological system and cytokine net in patients with malignant tumors. The elevated levels of TNF *α* in patients with malignant tumors may be also due to activation of angiogenesis and overexpression of TNF *α* in the transformed cells. Interferon γ is known as the most important factor to increase the expression of TNF *α*. The regulation of the expression is at the transcriptional level [27]. It has also been proven that elevated levels of TNF *α* may lead to overexpression of integrins and adhesive molecules [28]. Guardiola-Serrano *et al*. [29] assessed the influence of TNF *α* on the expression of ZC3H10 (zinc finger protein) and GRHL-3 (grainyhead-like 3) in breast cancer cell lines (MCF-7). Expression of these proteins was increased after addition of TNF *α* to the incubation medium of the cells *in vitro*. However, ZC3H10 acts as an antagonist of cell growth *in vitro*, but GRHL-3 stimulates the migration of the endothelial cells.

In our patients with malignant tumors of the adrenal cortex, in patients with Conn’s syndrome and in patients with non-functioning adenomas and pheochromocytomas the concentration of TNF *α* was decreased after adrenalectomy. These results may be due to elimination of the tumor, which was the main local source of TNF *α*.

The role of the soluble receptors of TNF *α* in the pathogenesis of adrenal tumors is unknown. Elevated levels of these receptors were found in the amniotic fluid of pregnant women [30,31], in the serum and plasma of patients with HIV [32] and patients with malignant tumors [33]. Some authors suggest that soluble forms of TNF *α* receptors may bind TNF *α* and inactivate this molecule. On the other hand, these forms of TNF *α* receptors can stabilize the TNF *α* molecule at low concentrations of TNF *α* [6].

The results of our study revealed that the concentration of soluble forms of TNF *α* receptors did not differ, either among study groups or compared to the control. After adrenalectomy, the levels of the soluble forms of TNF *α* decreased in patients with Conn’s syndrome. It could be argued that the soluble forms of TNF *α* receptors are also involved in the process of tumorigenesis. The low level of TNF *α* and high level of soluble forms of TNF *α* receptors in patients with myelolipoma may confirm the hypothesis that soluble forms of these receptors inactivate this molecule. However, such a small group (3 patients) is not sufficient to draw any significant conclusions.

To confirm practicality of the evaluation of TNF *α* and its soluble receptors in differential diagnosis in patients with adrenal tumors, a large study group is needed. The evaluation of the concentration of numerous cytokines in the blood has been used only in the clinical studies so far. To take advantage of these molecules in clinical practice, further studies are needed and clinical examinations, laboratory tests and imaging techniques will still be the most important means of diagnosing patients with adrenal tumors.

## 5. Conclusions

TNF *α* and its soluble receptors appear to be involved in adrenal gland oncogenesis. The peripheral blood concentrations of this cytokines before surgery may be useful for the discrimination between malignant and benign adrenal tumors.
